# An open access microfluidic device for the study of the physical limits of cancer cell deformation during migration in confined environments

**DOI:** 10.1016/j.mee.2015.02.022

**Published:** 2015-08-16

**Authors:** Majid Malboubi, Asier Jayo, Maddy Parsons, Guillaume Charras

**Affiliations:** a London Centre for Nanotechnology, University College London, UK; b Randall Division of Cell and Molecular Biophysics, Kings College London, UK; c Department of Cell and Developmental Biology, University College London, UK

**Keywords:** Microfluidics, Cell deformation, Breast cancer cells, Multilayer photolithography

## Abstract

During metastasis, cancerous cells leave the primary tumour, pass into the circulatory system, and invade into new tissues. To migrate through the wide variety of environments they encounter, the cells must be able to remodel their cell shape efficiently to squeeze through small gaps in the extracellular matrix or extravasate into the blood stream or lymphatic system. Several studies have shown that the nucleus is the main limiting factor to migration through small gaps (Wolf et al., 2013; Harada et al., 2014; Mak et al., 2013). To understand the physical limits of cancer cell translocation in confined environments, we have fabricated a microfluidic device to study their ability to adapt their nuclear and cellular shape when passing through small gaps. The device is open access for ease of use and enables examination of the effect of different levels of spatial confinement on cell behaviour and morphology simultaneously. The results show that increasing cell confinement decreases the ability of cells to translocate into small gaps and that cells cannot penetrate into the microchannels below a threshold cross-section.

## Highlights

• A microfluidic device to study cell and nuclear deformation during translocation.
• Adherent cells protrude their cytoplasm regardless of the channel cross-section.
• The nucleus acts as a limiting factor when the channel area is below 7 × 5 μm^2^.

## Graphical abstract

**Figure:**
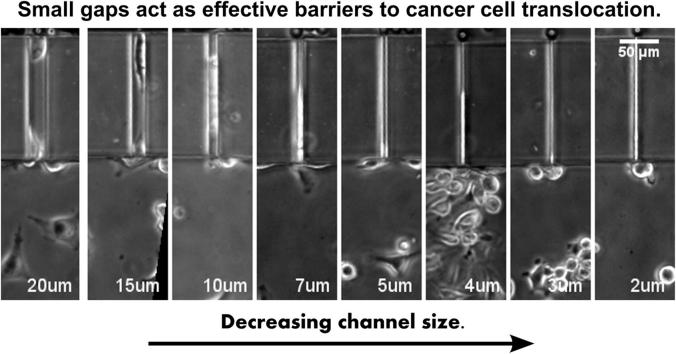

## 1 Introduction

**Fig. 1:**
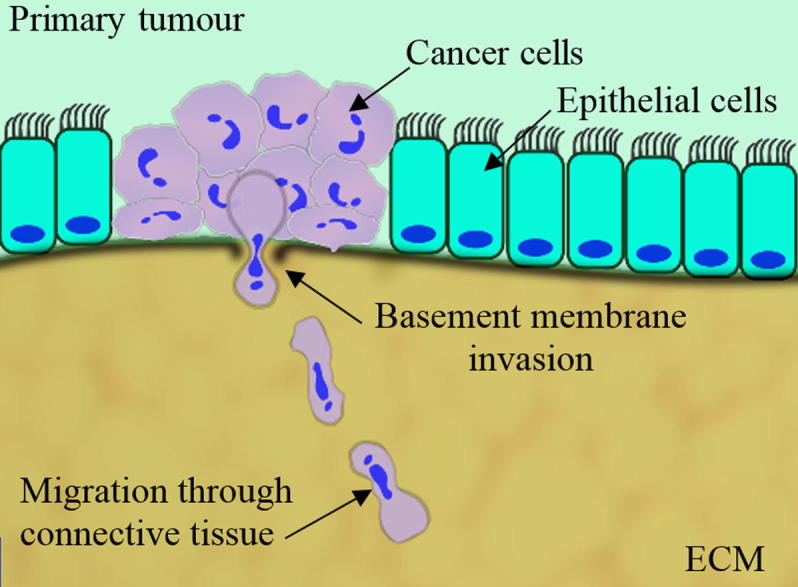
Early stages of metastasis formation and cancer cell invasion. During migration and invasion, cells must undergo large morphological changes in order to cross the basement membrane and move through connective tissue.

**Fig. 2:**
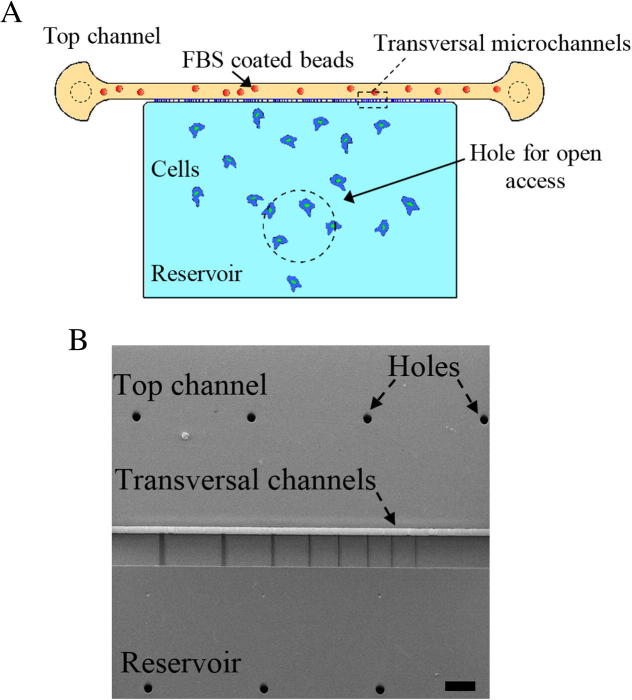
(A) Schematic of the microfluidic device. The area shown by the dashed-line is perforated to provide open access. (B) SEM image of the SU8 master mould, taken at an angle of 20° from the vertical to show the height difference between first and second layer of SU8. The channel height is 5 μm and the channel widths are 2, 3, 4, 5, 7, 10, 15 and 20 μm from left to right. The scale bar represents 100 μm.

Cell migration mediates a number of physiological and pathological processes and is an essential feature of cancer metastasis. During metastasis, cancer cells leave the primary tumour, extravasate into the blood stream, intravasate into new tissues, and migrate to form new colonies (Fig. 1). Throughout this process, they encounter many different extracellular environments and hence must show great plasticity in their migratory strategies. In particular, cancer cells are known to adapt their migratory strategies in response to extracellular cues in order to cross basement membranes and connective tissues [4]. During these processes, cells are confronted to different levels of physical confinement, moving across pores with a cross section ranging from 10 to 600 μm^2^ [5]. Being the largest and stiffest cellular organelle, nuclear deformation is a critical step during migration in confined environments [1,3]. Various microfluidic devices have been developed to study metastatic cell responses to physical confinements, chemical stimuli, or both simultaneously [3,6,7]. It has been shown that microfluidic channels with cross-sections smaller than the nuclear dimensions form effective barriers to prevent transmigration [8,9]. However, transmigration capabilities are cell-line specific and can be modulated by chemical treatments [3,6,7]. To study the effects of physical confinement in a non-pliable and non-degradable environment, we have developed microfluidic devices with arrays of micro-channels of different cross-sectional areas. MDA MB 231 human breast cancer cells were induced to migrate through the channels while the cellular and nuclear morphology was imaged. This allowed us to characterise the cells’ ability to adapt to different degrees of confinement and to study the deformation of the cytoplasm and the nucleus in conditions where proteolysis of the extracellular matrix can be disregarded. Fig. 2A shows a schematic of the device used in this study: the cells are loaded via an open access reservoir. A series of micro-channels connects this reservoir to a channel containing chemoattractant. Cellular and nuclear deformations are studied while cells translocate from the reservoir into the micro-channels.

## 2 Material and methods

### 2.1 Device design and fabrication

The device consists of an open access reservoir connected to a large channel by a series of transverse micro-channels. The micro-channels connecting the reservoir to the channel are 150 μm long, 5 μm high, and are arranged in groups with widths ranging from 2 to 20 μm (Fig. 2B). To ensure optimal bonding of the PDMS to the glass substrate, the distance between each pair of transversal micro-channels is ten times the width of the larger micro-channel. The total width of a set of transversal channels is 1 mm enabling imaging of all channels simultaneously in one field of view at 4× magnification.

Silicon wafer master moulds were manufactured using multilayer photolithography. The first layer consisted of transversal micro-channels with a height of 5 μm and was fabricated using a chrome mask and SU-8 2005. This layer was then aligned with a second transparency mask comprising the reservoir and the top channel. A height of 80 μm was chosen for the reservoir to allow enough space for cells to migrate without confinement and this was fabricated using SU8 2050. Because of the large overall dimensions of the top channel and the reservoir, pillars were included in this second layer to prevent the device from collapsing. PDMS was mixed with curing agent in a ratio of 10:1 and poured onto the mould. After curing at 65 °C, the device was peeled off from the mould. Holes were punched to provide an inlet and outlet for the top channel. The central part of reservoir was cut out with a biopsy punch to provide open access to the reservoir. The PDMS was subsequently bonded tightly onto glass bottom dishes using air plasma.

### 2.2 Cell culture

MDA MB 231 human breast carcinoma cells (ATCC) were cultured in high glucose DMEM supplemented with 10% Fetal Bovine Serum and Glutamine. For the study of the nuclear morphology, cells were transduced with lentiviruses to stably express RFP-lifeact and GFP-H2B constructs (Kind gifts from Dr. Tim Scales and Dr. James Monypenny, King’s College London, UK).

### 2.3 Experiments

Prior to the experiments, the chambers were coated with 10 μg/mL fibronectin in PBS injected through the top channel and incubated for 1 h at 37 °C. After coating, chambers were washed with 3 volumes of serum-free DMEM injected through the top channel. Cells were then trypsinised, re-suspended in serum-free medium containing 20 mM Hepes at 5 × 10^6^ cells/mL, loaded into the reservoir chamber using a regular micropipette and were left to spread for 3 h. To obtain a gradual delivery of chemoattractants and stable gradient formation without flow, 15 μm diameter polystyrene beads (Polysciences, Eppelheim, Germany) were coated with Fetal Bovine Serum (FBS). Serum-coated beads were injected into the chemoattractant channel, left to rest during 30 min to allow gradient formation and flow stabilisation, and the chamber was placed on the microscope stage for live cell imaging. During the experiments dishes, were filled with sufficient culture medium to prevent evaporation. In some experiments, to visualise gradient formation, beads were coated with Rhodamine Isothiocyanate and FBS, washed several times, and imaged immediately after bead injection into the upper channel.

### 2.4 Live cell imaging

Live cell imaging was performed in an Olympus IX71 wide-field epi-fluorescence microscope with an incubation chamber (Olympus, Tokyo, Japan), attached to an Andor iXON EMCCD camera (Belfast, UK). Images were acquired at 10 min intervals for 10 h, using a 20× magnification 0.4 numerical aperture air objective. All the image analysis was performed using ImageJ software (NIH, Bethesda, USA).

### 2.5 Statistical analysis

Nuclear translocation was quantified as the percentage of cells that inserted completely their nuclei inside the microchannel from the total number of cells that were able to protrude their cytoplasm into it. Microchannel area was defined by phase contrast image and nuclear area by the H2KB-GFP signal. Differences in translocation for different channel widths were tested using a one-way ANOVA and *post hoc* Least Significance Difference (LSD) test using *p* < 0.05 as a significance threshold with the SPSS software (IBM Corp., Armonk, NY, USA). Curve fit was performed using Origin 9.0 software (OriginLab, MA, USA).

## 3 Result and discussion

**Fig. 3:**
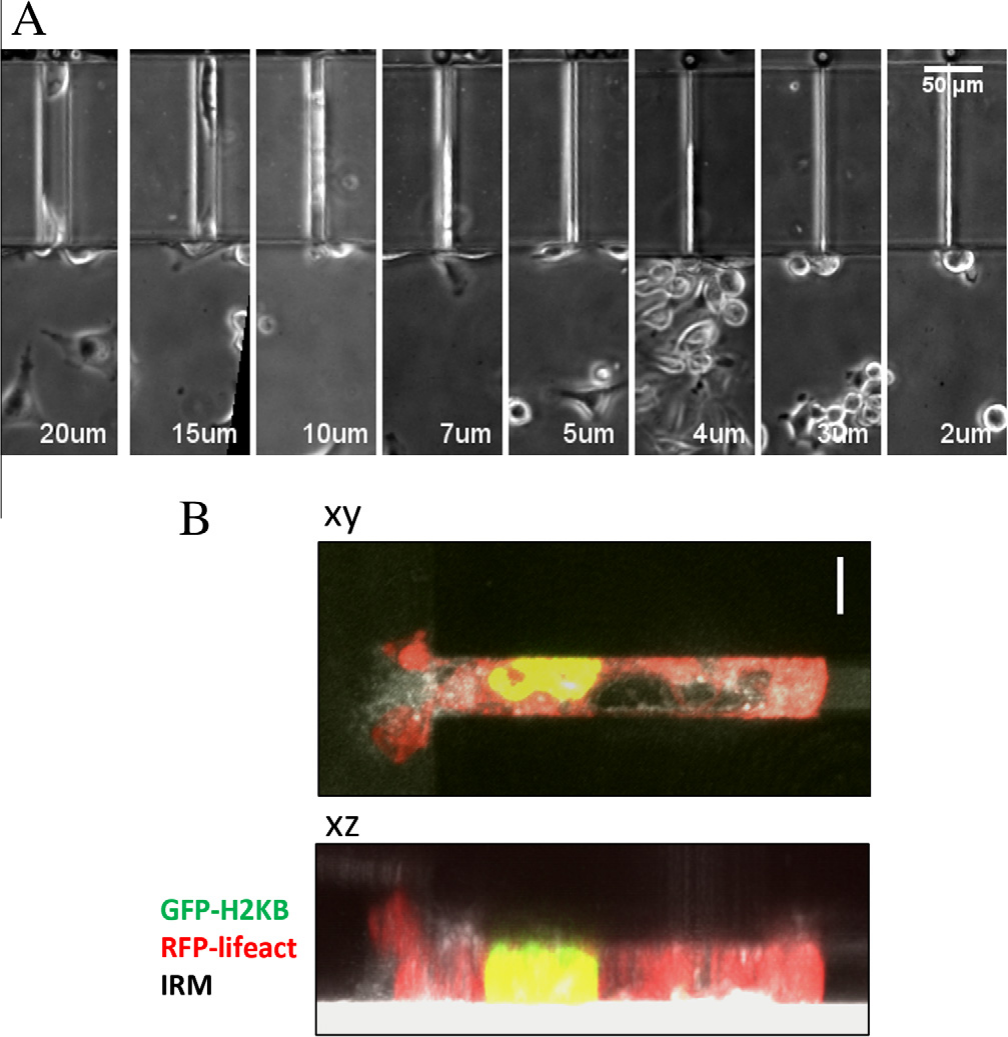
(A) Representative phase contrast images of cells migrating through channels with widths of 20–2 μm. Cells are able to protrude into the channels irrespective of their width. (B) Maximum intensity projections of a cell migrating through a 10 μm-width microchannel. GFP-H2KB, RFP-lifeact, and reflection are shown in green, red, and grayscale, respectively. Scale bar: 10 μm. (For interpretation of the references to colour in this figure legend, the reader is referred to the web version of this article.)

To induce cancer cell directional migration and translocation into the microchannels, a chemotactic gradient of FBS was generated by injecting polystyrene beads coated with FBS in the top channel. This allows a gradual delivery and stable gradient formation without flow and gradients can be visualised by co-coating the beads with fluorescent trackers [7]. Fig. S1 shows that the gradient formation starts immediately after bead injection and that the gradient stabilises after one hour, remaining stable for at least 8 h. Over the duration of the experiments, MDA MB 231 cells were able to insert their cell front inside the micro-channels in response to the FBS gradient, irrespective of the micro-channel width (Movie S1). Fig. 3A shows a representative snapshot of MDA MB 231 cells migrating through the transversal channels, where each cell faces a different level of spatial confinement. Confocal imaging of cells migrating through the microchannels showed that the 5 μm height of the channel limits itself the height of the cell, regardless of the width, and that the nuclei occupies the whole height of the channel (Fig. 3B).

In our system, cell protrusion rate can be monitored by phase contrast microscopy and the cell profiles over time can be plotted in a kymograph, as shown in Fig. S2A. This approach showed that cell progression through the micro-channels is limited by their cross-section, with widths smaller than 5 μm largely impeding cell migration.

**Fig. 4:**
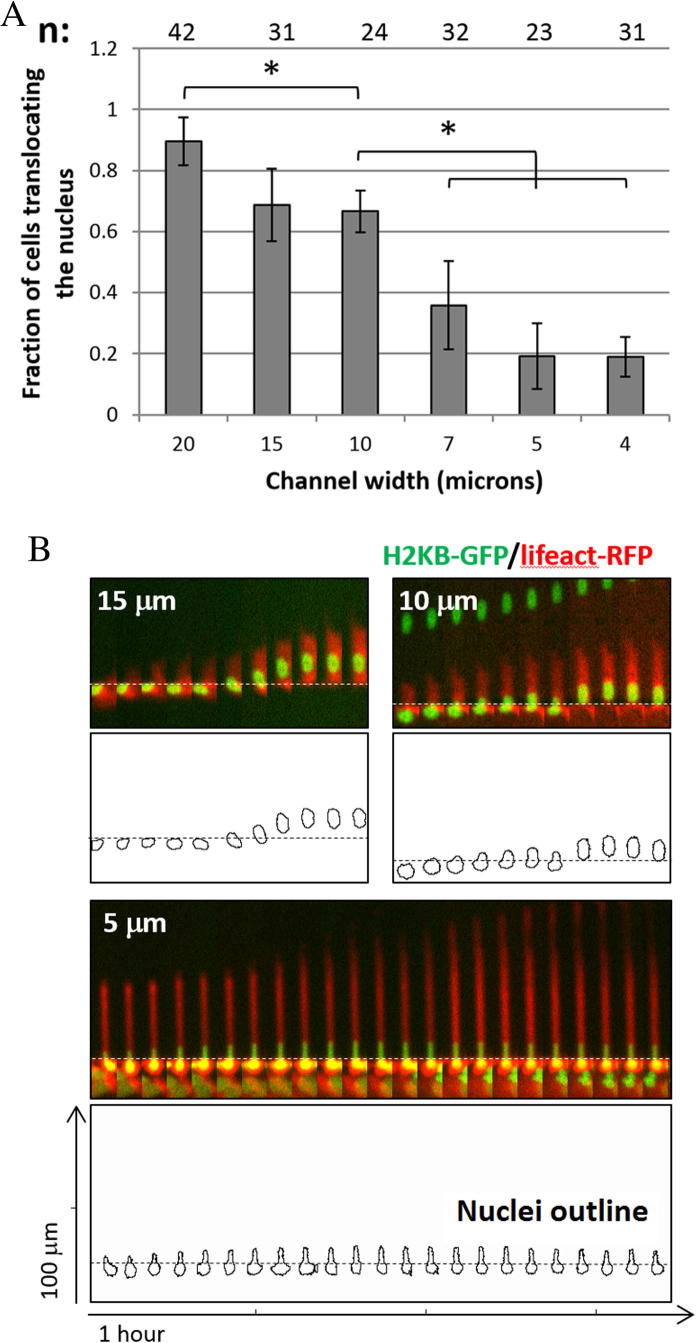
(A) Quantification of the ratio of cells trans-locating their nucleus inside a channel to the cells protruding into the channels. *n* = number of events quantified in each condition in three independent experiments. ^∗^*p* < 0.05 (B) Kymographs of representative single cells translocating their nuclei into channels of different width. The upper panel shows cells expressing GFP-H2B (green) and RFP-lifeact (red). The lower panel shows the outline of the nuclei. Dashed lines indicate the entrance to the channels. (For interpretation of the references to colour in this figure legend, the reader is referred to the web version of this article.)

In confined environments, it is generally assumed that the nucleus, being the biggest and stiffest organelle, is the rate limiting factor to invasion [10]. Several studies have recently attracted attention towards the role of the nucleus and its physical properties in cell migration in three-dimensional, spatially confined environments [1,2,11]. By using our microfluidic device together with cells expressing nuclear and cytoskeletal markers, we were able to study in real time the behaviour of cells during the translocation of their nucleus into the microchannels. Initially, we quantified the proportion of cells trans-locating their nucleus into the microchannels. As shown in Fig. 4A, nuclear translocation though pores with a cross-section below 7 × 5 μm^2^ was significantly impaired compared to wider channels. The relation between cells’ ability to translocate and spatial constriction, fitted with a sigmoid curve presented an inflection point for a 8.3 × 5 μm cross-section (Fig. S2B). In our experiments, this threshold was apparent for 7 μm width microchannels, probably reflects MDA-MB-231e cells’ physical limit in a non-pliable and non-degradable, confined environment. This was consistent with previously published results, which found that channels with cross sections of 6 × 5 μm^2^ and 4 × 5 μm^2^ considerably reduce transmigration of MDA-MB-231 and MCF7 cell lines respectively [1,8,9].

Our system also allows the study of cell protrusion and nuclear area in real-time during nuclear translocation. In Fig. 4B, kymographs show the profile of the cell and nuclear outline as well as the evolution of nuclear shape and area during this process. This data can later be used to study how cell protrusion rates can correlate with nuclear displacement and nuclear area changes during the translocation process, and how these ratios can change under different levels of spatial challenge (Fig. S3). More in depth analysis of this data could eventually help us understand the regulation of cell cytoskeleton and nuclear morphology during cancer cell translocation to confined environments.

## 4 Conclusion

Here we present a new, open access, microfluidic device for the study of cell and nuclear deformation during translocation into spatially confined environments. Our results show that adherent cells can protrude their cytoplasm regardless of the channel width in response to chemoattractant, while the nucleus acts as a limiting factor in the whole cell displacement when the channel cross-section is below 7 × 5 μm^2^, in agreement with previous studies. Additionally, our system allows real-time imaging of cell and nuclear morphology during translocation, which makes it very suitable for the study of cells’ ability to adapt to confined environments.

Deeper analysis performed on the data acquired with this device will provide us with new insights into the ability and molecular mechanisms that cancer cells use in order to adapt to their extracellular environment during migration.

## Appendix A Supplementary data

Supplementary data 1 This document contains supplementary figures.
