# Supplementary material for: An open access microfluidic device for the study of the physical limits of cancer cell deformation during migration in confined environments
Source: Microelectron Eng. 2015 Aug 16;144:42–5. doi: 10.1016/j.mee.2015.02.022 (PMC4567073; doi:10.1016/j.mee.2015.02.022)
Supplement: Supplementary data 1 — This document contains supplementary figures. [file mmc1.pdf]

Supplementary figures

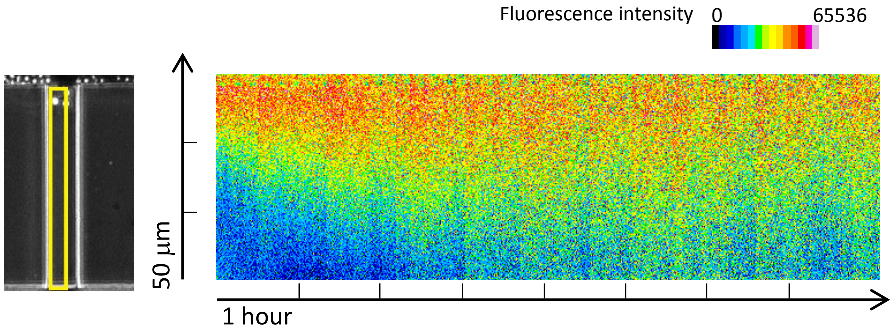

Figure S1. Right hand side panel shows the area highlighted in left hand side panel during the experiment. In the kymograph, Rhodamine fluorescence is visualised and pseudocolored with a 16 colours ramp. Gradient formation occurs during the first hour of the experiment and it remains stable for the next 8 hours.

A.

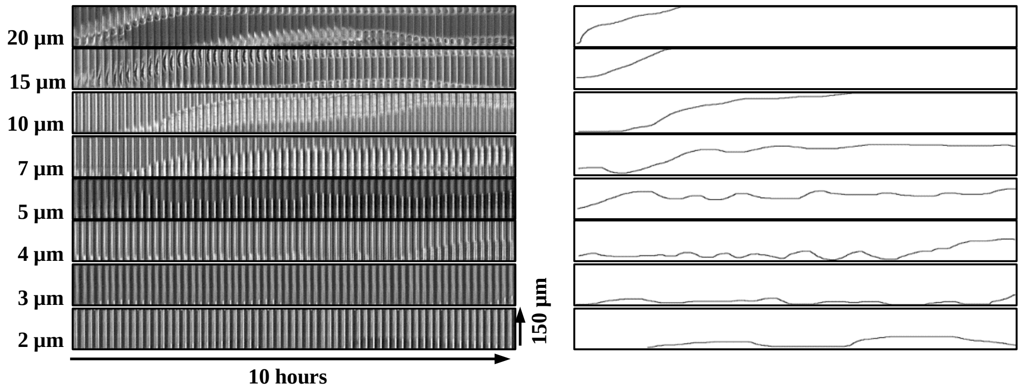

B.

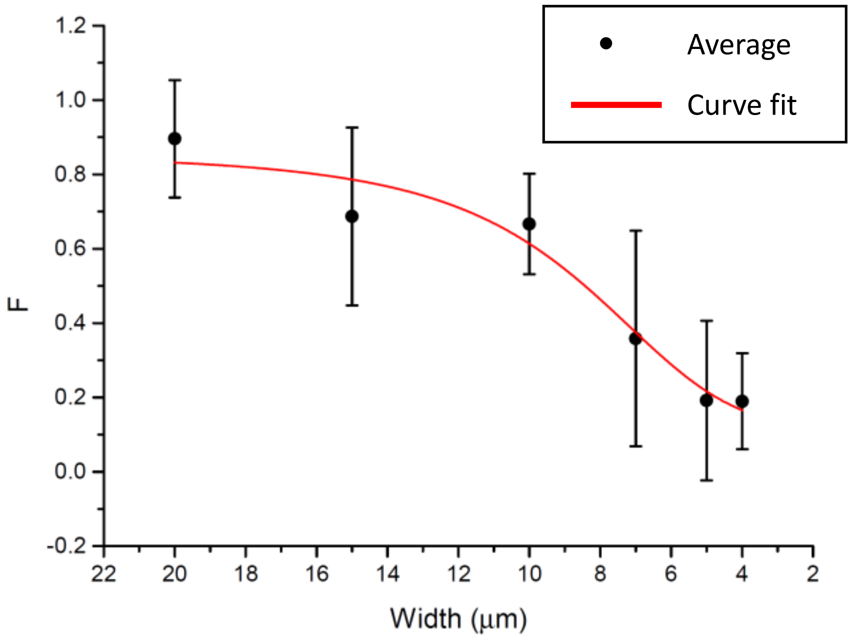

Figure S2. (A) Left panel: Representative kymographs of cells protruding through channels with different width over 10 hours. Right: Binary representation of cell front. (B) Logistic fit of the fraction of cells trans-locating their nucleus inside a channel versus microchannel width.

Y = A2 + (A1-A2) / [1 + (x/xo) ^ p]

Where A1 = 0.12231, A2 = 0.58733, x0 = 8.30664, and p = 3.77964. R-square = 0.8945.

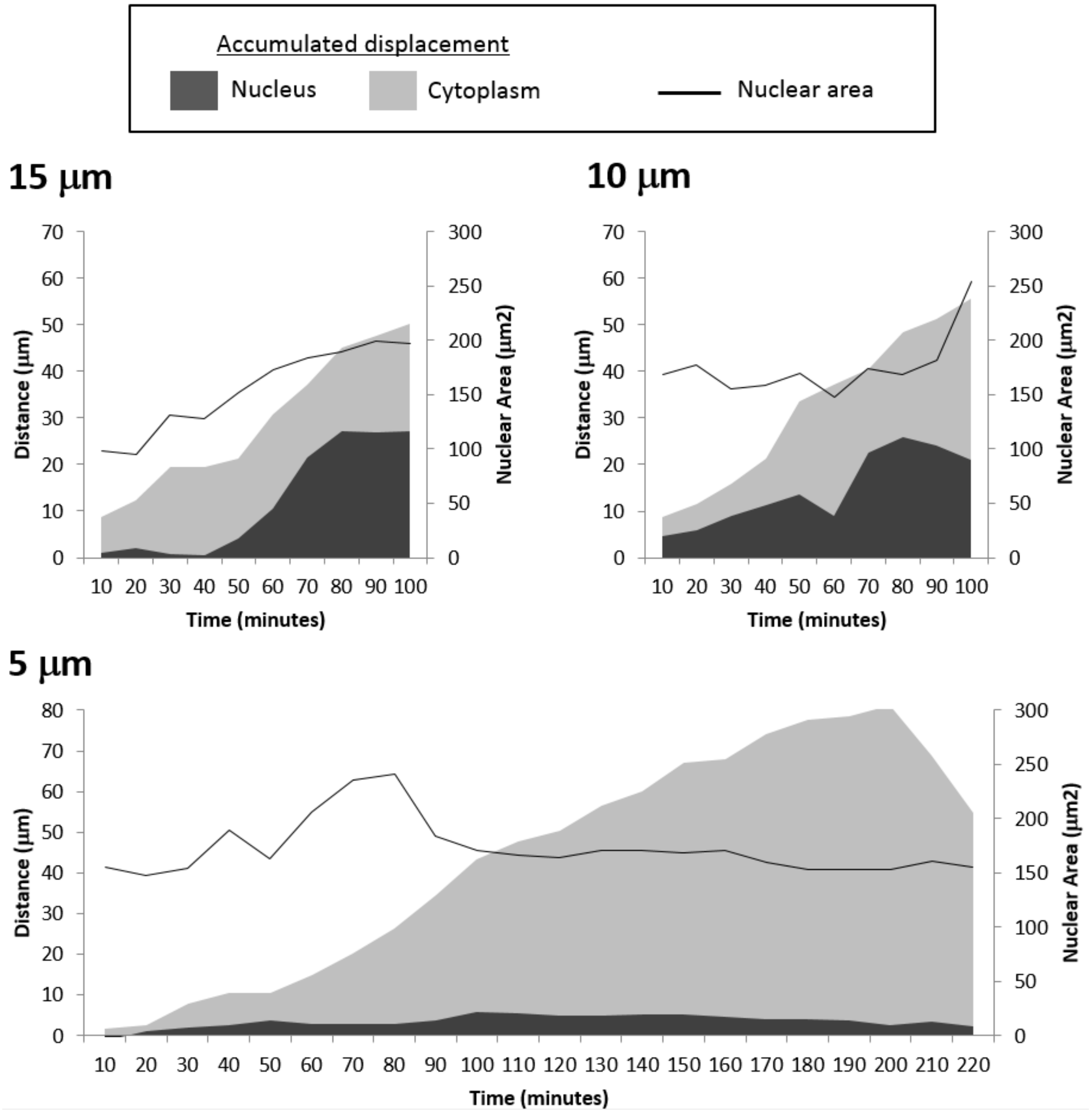

Figure S3. Analysis of representative cell translocation events shown in Figure 4B. Cell front (light grey coloured area) and nuclear centroid's (dark grey coloured area) were plotted with nuclear area changes (line graph).
