# Supplementary figures and images for: An open access microfluidic device for the study of the physical limits of cancer cell deformation during migration in confined environments
Source: Microelectron Eng. 2015 Aug 16;144:42–5. doi: 10.1016/j.mee.2015.02.022 (PMC4567073; doi:10.1016/j.mee.2015.02.022)

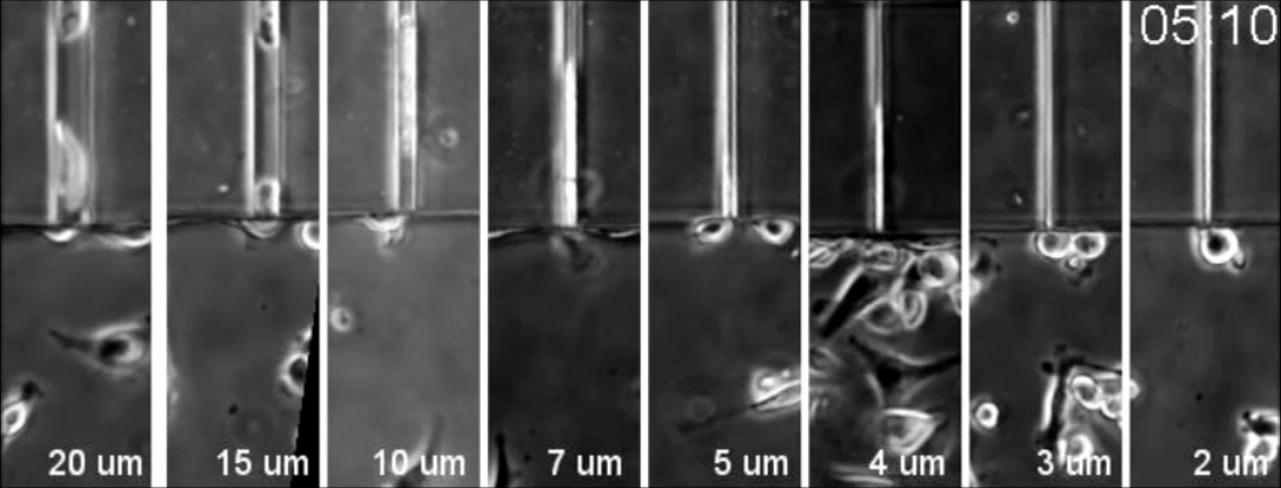

Supplement: Supplementary video 2 [file mmc2.jpg]
